# Refining Prognosis in Localized Gastrointestinal Stromal Tumor: Clinical Significance of Phosphatase and Tensin Homolog Low Expression and Gene Loss

**DOI:** 10.1200/PO.22.00129

**Published:** 2022-08-24

**Authors:** Xiaolan Feng, Haocheng Li, Joanna Fourquet, Mehdi Brahmi, Armelle Dufresne, Alexandra Meurgey, Isabelle Ray-Coquard, Qing Wang, Julien Bollard, Francoise Ducimetiere, Frederic Chibon, Jean-Yves Blay

**Affiliations:** ^1^ Department of Medicine Oncology, Tom Baker Cancer Center, Calgary, Alberta, Canada; ^2^ Cumming School of Medicine, University of Calgary, Calgary, Alberta, Canada; ^3^ Department of Medicine, University of British Columbia, Vancouver, British Columbia, Canada; ^4^ Department of Mathematics and Statistics, University of Calgary, Calgary, Alberta, Canada; ^5^ OncoSarc, INSERM U1037, Cancer Research Center in Toulouse, Toulouse, France; ^6^ Department of Medical Oncology, Centre Léon Bérard, Lyon, France; ^7^ Department of Pathology, Institut Claudius Régaud, IUCT-Oncopole, Toulouse, France; ^8^ Department of Biopathology, Centre Léon Bérard, Lyon, France; ^9^ University Claude Bernard Lyon, Lyon, France; ^10^ Department of Translational Research and Innovation, Centre Léon Bérard, Lyon, France

## Abstract

To investigate the use of PTEN biomarker to improve prognostic stratification in patients with localized gastrointestinal stromal tumor (GIST).

**METHODS:**

PTEN expression and genomic analysis were performed on two independent GIST-60 (n = 60) and GIST-100 (n = 100) cohorts, respectively.

**RESULTS:**

PTEN expression was significantly lower in patients with local and metastatic recurrent tumor compared with those with no recurrence (*P* = .004). PTEN low expression was significantly associated with poor disease-free survival (DFS) compared with PTEN high expression (43.73 *v* 117.95 months; *P* = .0084) and distant metastatic-free survival (DMFS; 57.95 *v* 117.95 months; *P* = .0032). PTEN heterozygous loss was observed in approximately 10% of the patients in each cohort and was associated with poor DFS compared with patients with PTEN normal status (27.56 months *v* not reached [NR]; *P* < .001) and DMFS (27.56 months *v* NR; *P* < .001). Multivariate analysis revealed that PTEN expression was an independent clinical prognosis factor besides tumor size, mitosis index, and location (hazard ratio for DFS: 3.8; *P* = .033; hazard ratio for DMFS 5.7, *P* = .01). Furthermore, PTEN low expression was independently associated with poor DMFS in clinically high-risk patients (mDMFS: 42.28 *v* 65.61 months; *P* = .0166). In addition, PTEN heterozygous loss was independently associated with poor DMFS in patients at either low/intermediate risk (mDMFS: 18.05 months for *PTEN* loss *v* NR for PTEN normal status; *P* < .001) or at high risk (mDMFS: 27.19 months for PTEN loss *v* 105.36 months for PTEN normal status; *P* = .044).

**CONCLUSION:**

PTEN low expression/gene loss is an independent significant prognostic factor and a promising component to strengthen the clinical prognostic tools in patients with localized GIST.

## INTRODUCTION

Gastrointestinal stromal tumors (GISTs) are the most common mesenchymal malignancies of the gastrointestinal tract. As tumors driven by KIT or PDGFRA oncogenic mutations, patients with GIST clearly benefit from the development of molecularly targeted treatments.^1^ For early-stage/localized GIST amenable to resection, several clinical prognostic factors have been identified and prospectively validated to guide clinical management in terms of surveillance and help decision in guiding adjuvant treatment.^2-7^ The current clinical factors include age, tumor size, mitotic count, location, and perforation. To date, there is no consistent and validated prognostic biomarker routinely used in patients with resected GIST other than mutation status in receptor tyrosine kinases (RTKs) KIT or PDGFRA, likely because of the lack of in-depth understanding in the biological mechanisms of disease relapse beyond kinase-activating mutations. Despite effective targeted adjuvant treatment with imatinib is proposed in patients who are clinically considered at high risk on the basis of standardized and validated clinical prognostic tools,^2-7^ about 35% patients still relapse.^8^ In addition, imatinib is currently only approved for patients with high-risk disease; about 5%-20% in patients with low to intermediate risk will relapse with or without imatinib.^2-7,9,10^

**CONTEXT**

**Key Objective**
To date, there is no consistent and validated prognostic biomarker routinely used in patients with resected gastrointestinal stromal tumor other than mutation status in receptor tyrosine kinases (KIT or PDGFRA), likely because of the lack of in-depth understanding in the biological mechanisms of disease relapse beyond kinase-activating mutations. This study is to investigate the use of Phosphatase and TENsin homolog deleted on chromosome 10 (PTEN) biomarker to improve prognostic stratification in patients with localized gastrointestinal stromal tumor.
**Knowledge Generated**
PTEN low expression and PTEN loss consistently and independently predicted poorer survival in clinically high-risk patients but also in patients at low/intermediate risk.
**Relevance**
Use of a further validated PTEN immunohistochemistry assay (PTEN low expression/loss) could allow stratification of clinically high-risk patients to participate in clinical trials to investigate the additional value of mTOR inhibitors to standard adjuvant imatinib, and clinically low-/intermediate-risk patients to undergo intense surveillance.

Phosphatase and TENsin homolog deleted on chromosome 10 (PTEN) is a tumor suppressor known as one of the central regulators of phosphatidylinositol-3-kinase**/**protein kinase B/mammalian target of rapamycin (PI3K/Akt/mTOR) pathway, involved downstream RTKs. PTEN loss of function leads to upregulation of the pathway that stimulates cell growth and survival.^11^ In addition, PTEN loss and activation of PI3K/Akt/mTOR pathway is more frequently seen in advanced GIST or imatinib-resistant GIST tumors, underlining its critical role in promoting tumor progression and conferring resistance to inhibitors of RTKs.^12-14^ Loss of PTEN expression is observed in 38.6% of soft tissue sarcoma (STS), most commonly in leiomyosarcomas, epithelioid sarcomas, alveolar rhabdomyosarcomas, osteosarcomas, and chordomas.^15^ The mutations and deletions in PTEN occur in 2%-10% of STS.^16^ In GIST, limited studies published to date with sample sizes between 20 to just over 100 revealed that around 10%-50% of primary/untreated GISTs with either PTEN low expression or PTEN loss are associated with high-risk tumors and unfavorable clinical outcomes.^12,17-19^ None of these studies specifically reported the use of PTEN biomarker to strengthen the current clinically standardized prognostic factors in patients with GIST. Clinical studies investigating mTOR inhibitors as monotherapy in many STS including GISTs did not show significant efficacy assuming a negative feedback loop activation of AKT and mTOR complex 2 (mTORC2) in addition to mitogen-activated protein kinase (MAPK) pathway.^20,21^ Several preclinical studies showed that combined treatment with imatinib and PI3K inhibitors were more effective than imatinib as single agent.^22-24^ Thus far, only one phase I/II study, to our knowledge, revealed some activity with the combination of imatinib and the mTOR inhibitor everolimus in patients who progressed with imatinib or sunitinib alone.^25^ The results from ongoing trials (ClinicalTrials.gov identifier: NCT01735968, NCT01468688, NCT00087074) are still pending.

This study investigates whether PTEN biomarker added an independent prognostic value to current standardized clinical prognostic tools in patients with localized GIST. PTEN biomarker may help to improve clinical management of resected GIST patients (ie, increase surveillance) and may be used as stratification factor in adjuvant clinical trials dedicated to patients with GIST to further investigate combined treatments with TKIs and mTOR inhibitors and contribute to improve clinical outcome in patients with early-stage GIST.

## METHODS

### Patient/Tumor Samples

This retrospective study enrolled patients with localized GIST diagnosed from June 1995 to February 2009 and confirmed by a central histologic review according to the French Sarcoma Group (FSG) guidelines. All tumor samples are recorded in the European GIST database (ConticaGIST)^26^ under the umbrella of ATGsarc database^27^ (on-demand access).^28,29^ Frozen tumor samples were obtained from primary tumor resection. GIST sample classification used the modified National Institute of Health (NIH)^28,29^ and American Forces Institute of Pathology (AFIP) prognostic criteria and prognostic contour map.^2,3,6^

### Gene Expression and Comparative Genomic Hybridization Analysis

PTEN expression and comparative genomic hybridization (CGH) analysis were carried out using 44K (model 014850, Agilent Technology, Santa Clara, CA) and 8 × 60K whole-genome Agilent arrays (model G4450A, Agilent Technology), respectively, according to the manufacturer's protocol. Gene expression analysis was performed on GIST-60 cohort, whereas CGH analysis was conducted on GIST-100 cohort as previously detailed.^29^ The probe A_24_P913115 maximizing the interquartile range value (ie, higher dispersion) was selected to reflect PTEN expression. In CGH analysis, PTEN heterozygous and homozygous loss were defined as the absence of a single and both PTEN copies, respectively, whereas PTEN gain and amplification were defined as the presence of 2 to 10, and more than 10 gene copies, respectively. Data are available online in the ATGsarc database upon request.

### Statistics

Descriptive analysis for patient and tumor characteristics were presented in the two cohorts (GIST-60, n = 60; GIST-100, n = 100). Box plots illustrate PTEN expression levels in patients with or without local or metastatic recurrence, and the Wilcoxon nonparametric test was used to compare PTEN expression levels between groups. The mean PTEN expression was selected as cutoff to differentiate low and high PTEN expression in the GIST-60 cohort. Indeed, previous reports showed that 50% of the GISTs have PTEN low expression or PTEN gene loss.^12,19^ Disease-free survival (DFS) and distant metastasis-free survival (DMFS) estimates were calculated using the Kaplan-Meier (KM) method. Subgroup comparisons were performed using log-rank tests. Median follow-up (FU) was calculated using reverse Kaplan-Meier estimation. Cox proportional hazard model was used to identify the prognostic value of PTEN on DFS and DMFS. The multivariate model included known clinical prognostic factors such as tumor size, mitotic count, and location. Hazard ratios (HRs) are presented with 95% CIs. All statistical analyses were performed using the software program R v4.1.2 (R Foundation for Statistical Computing, Vienna, Austria).^30^

### Ethics Approval

Data collection and analysis received approval from the ethics committees according to applicable national legislation, authorization from Comité consultatif sur le traitement de l'information en matière de recherche dans le domaine de la santé (CCTIRS) number 09.594 received on November 19, 2009, and authorization from the Comité National Informatique et Liberté (CNIL) number 909510, received on February 5, 2010.

## RESULTS

### Cohort Characteristics

Patient and tumor characteristics of the GIST-60 (n = 60) and GIST-100 (n = 100) cohorts are summarized in Table 1. The median age at diagnosis was 62 years (range, 36-76 years) and 64 years (range, 29-86 years) in GIST-60 and GIST-100 cohorts, respectively. The majority of tumors were located in stomach (GIST-60: n = 40, 66.7%; GIST-100: n = 48; 48%) and small bowel (GIST-60: n = 14, 23.3%; GIST-100: n = 26, 26%). A minority of tumors had size > 10 cm (GIST-60: n = 12, 20%; GIST-100: n = 14, 14%) and mitotic rate over 10/50 high-power field (GIST-60: n = 13, 21.7%; GIST-100: n = 10, 10%). Just over a third of the patients (GIST-60: n = 23, 38.3%; GIST-100: n = 35, 35%) were classified at high risk on the basis of modified NIH criteria. Less patients at high risk were identified according to the AFIP prognostic criteria/prognostic contour map, also considering unknown tumor rupture status (GIST-60: n = 13, 21.7%; GIST-100: n = 9, 9%; Table 1). Patients had not received TKIs such as imatinib before or after surgical resection unless they developed distant metastasis. Median FU was 43.23 months (95% CI, 35.75 to 53.16) in the GIST-60 cohort and 50.46 months (95% CI, 41.74 to 59.50) in the GIST-100 cohort.

**TABLE 1.**
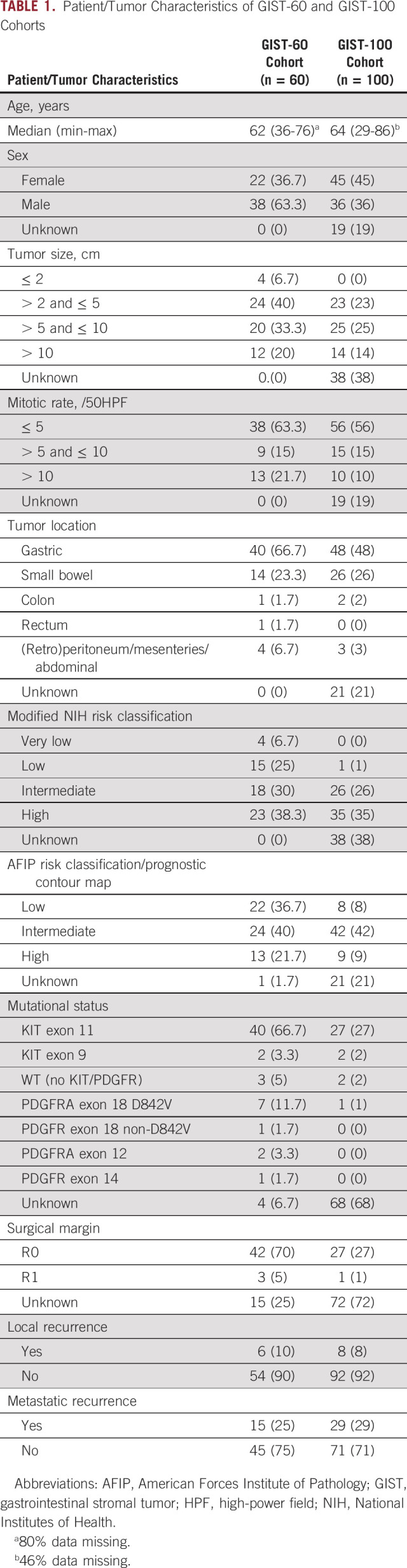
Patient/Tumor Characteristics of GIST-60 and GIST-100 Cohorts

### PTEN Expression and CGH Analysis

**FIG 1.**
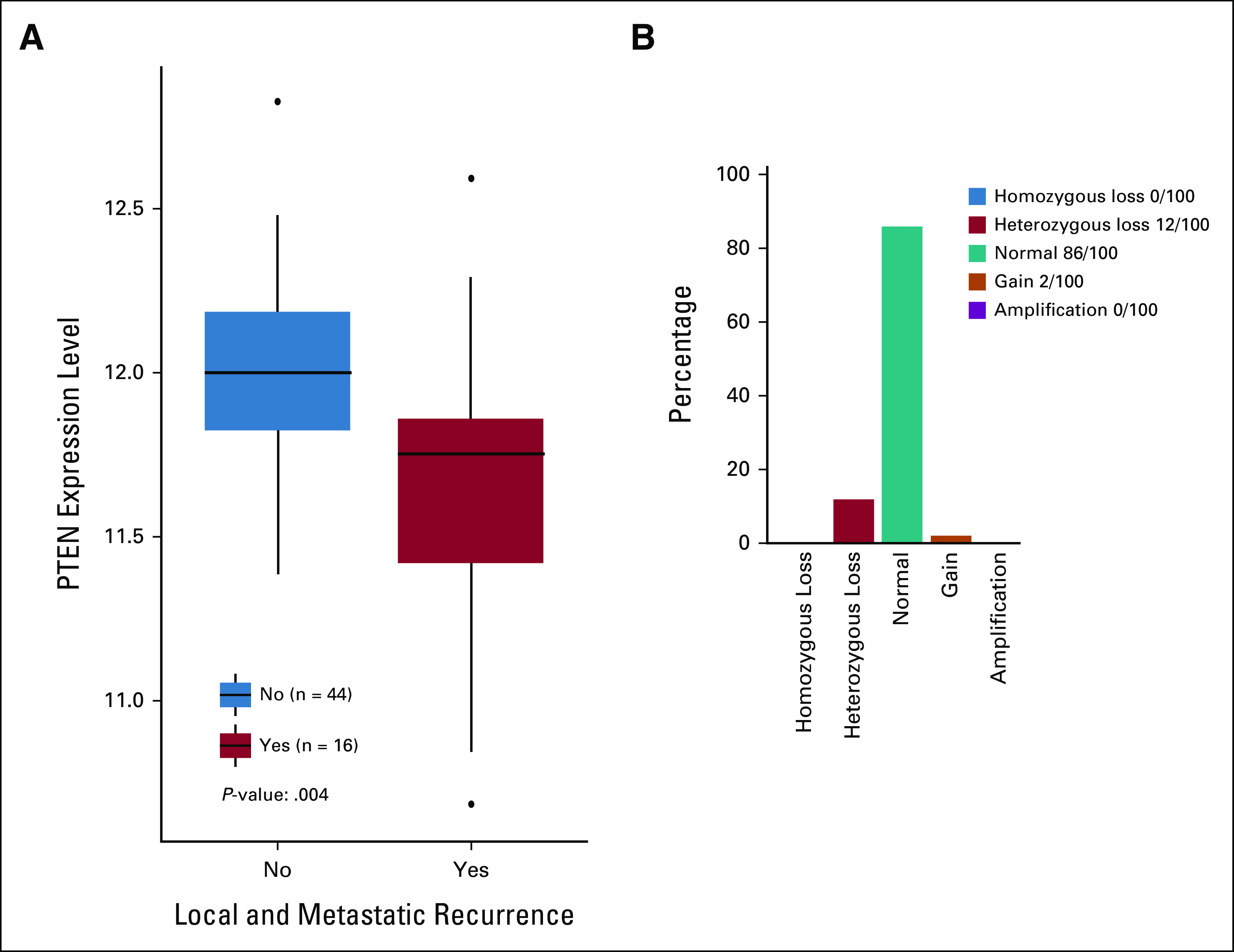
Lower expression and heterozygous Loss of PTEN in recurrent and metastatic GIST: (A) PTEN expression in the GIST-60 cohort and (B) heterozygous loss of PTEN in the GIST-100 cohort. GIST, gastrointestinal stromal tumor.

The transcriptional profiling in the GIST-60 cohort revealed a significantly lower PTEN expression in patients with local and metastatic relapse (n = 16) compared with those with no documented recurrence (n = 44; *P* = .0004; Fig 1A). CGH analysis revealed PTEN heterozygous loss in 12 (12%) in the GIST-100 cohort (Fig 1B).

### The Lower Expression and Heterozygous Loss of PTEN Predicted Poorer Survival

Survival analyses were performed in patients with PTEN low or high expression levels in the GIST-60 cohort and PTEN heterozygous loss or normal status in the GIST-100 cohort.

**FIG 2.**
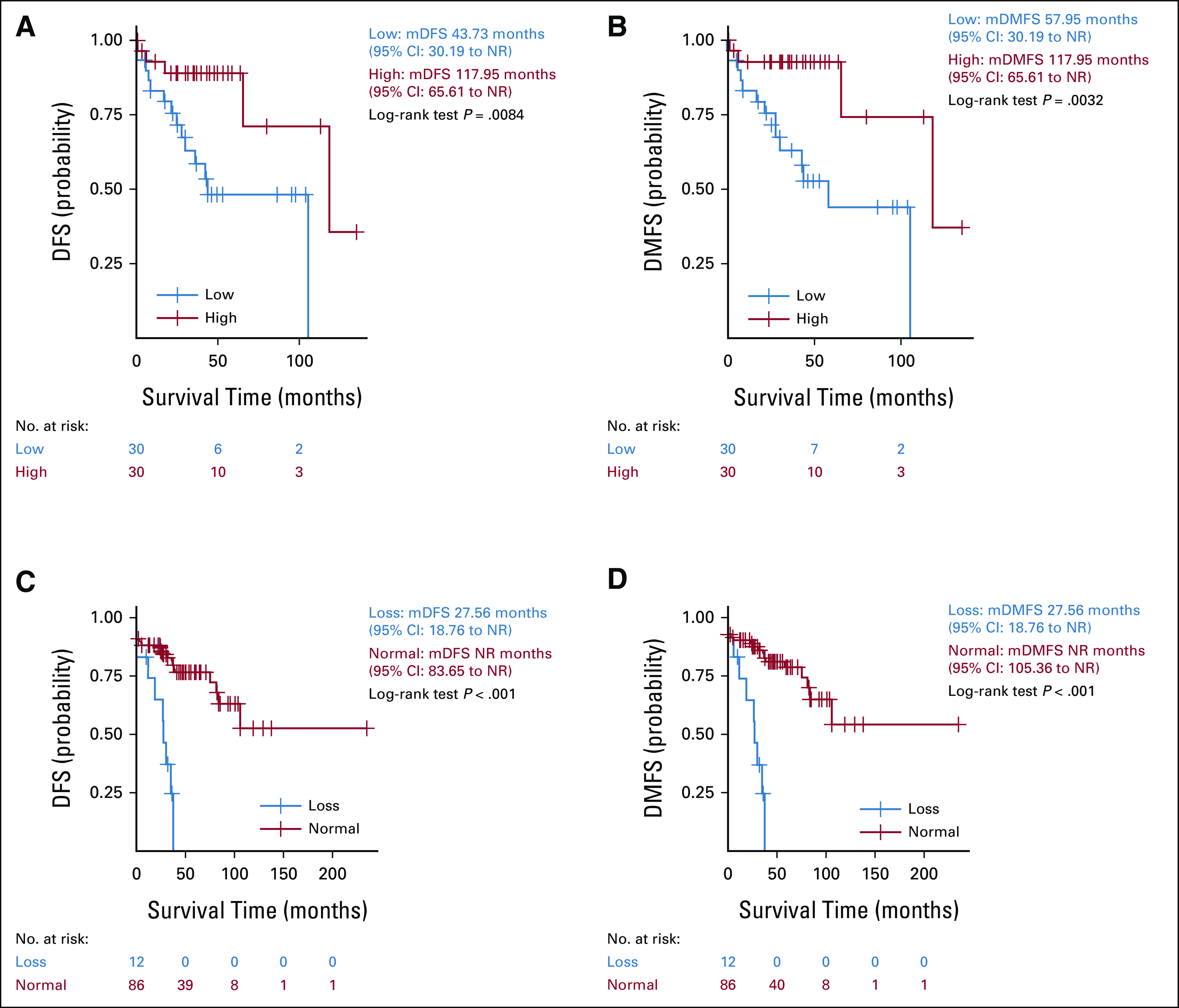
Low expression and heterozygous loss of PTEN predict poor disease-free survival (DFS) and DMFS in GIST: (A) DFS; GIST-60 cohort, (B) DMFS; GIST-60 cohort, (C) DFS; GIST-100 cohort, and (D) DMFS; GIST-100 cohort. GIST, gastrointestinal stromal tumor; DFS, disease-free survival; DMFS, distant metastatic-free survival; NR, not reached; CI, confidence interval.

In the GIST-60 cohort, patients with PTEN low expression had significantly reduced DFS (mDFS, 43.73 months; 95% CI, 30.19 to not reached [NR]) compared with patients with PTEN high expression (mDFS, 117.95 months; 95% CI, 65.61 to NR; *P* = .0084). Similarly, patients with PTEN low expression had a distant metastatic-free survival (DMFS) significantly reduced (mDMFS, 57.95 months; 95% CI, 30.19 to NR) compared with patients with high PTEN expression (mDMFS, 117.95 months; 95% CI, 65.61 to NR; *P* = .0032; Figs 2A and 2B). In GIST-100, patients with PTEN heterozygous loss had significantly poorer DFS (mDFS, 27.56 months; 95% CI, 18.76 to NR) compared with patients with PTEN normal status (mDFS, NR; 95% CI, 83.65 to NR; *P* < .001). Similarly, patients with PTEN heterozygous loss had significantly poorer DMFS (mDMFS, 27.56 months; 95% CI, 18.76 to NR) than patients with PTEN normal status (mDMFS, NR; 95% CI, 105.36 to NR; *P* < .001; Figs 2C and 2D).

Identification of PTEN low expression/genomic loss as an independent prognostic factor strengthens the clinical prognostic tools.

The significant prognostic value of PTEN in localized GIST was used to evaluate whether PTEN low expression provides independent additional value to already validated clinical prognostic factors. The multivariate analysis in GIST-60 revealed significant prognostic value of PTEN low expression (HR for DFS 3.8; *P* = .033; HR for DMFS 5.7, *P* = .01; Table 2). PTEN low expression is identified as an independent factor that may be added to already identified prognostic factors used at clinical level, ie, tumor size, mitotic count, and location.

**TABLE 2.**
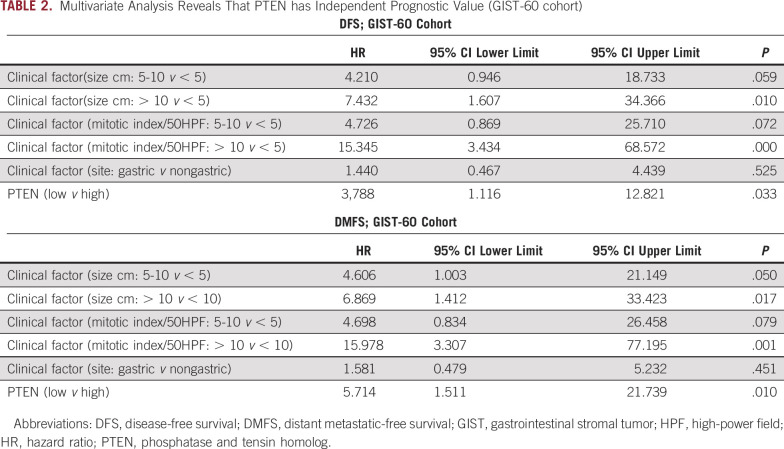
Multivariate Analysis Reveals That PTEN has Independent Prognostic Value (GIST-60 cohort)

**FIG 3.**
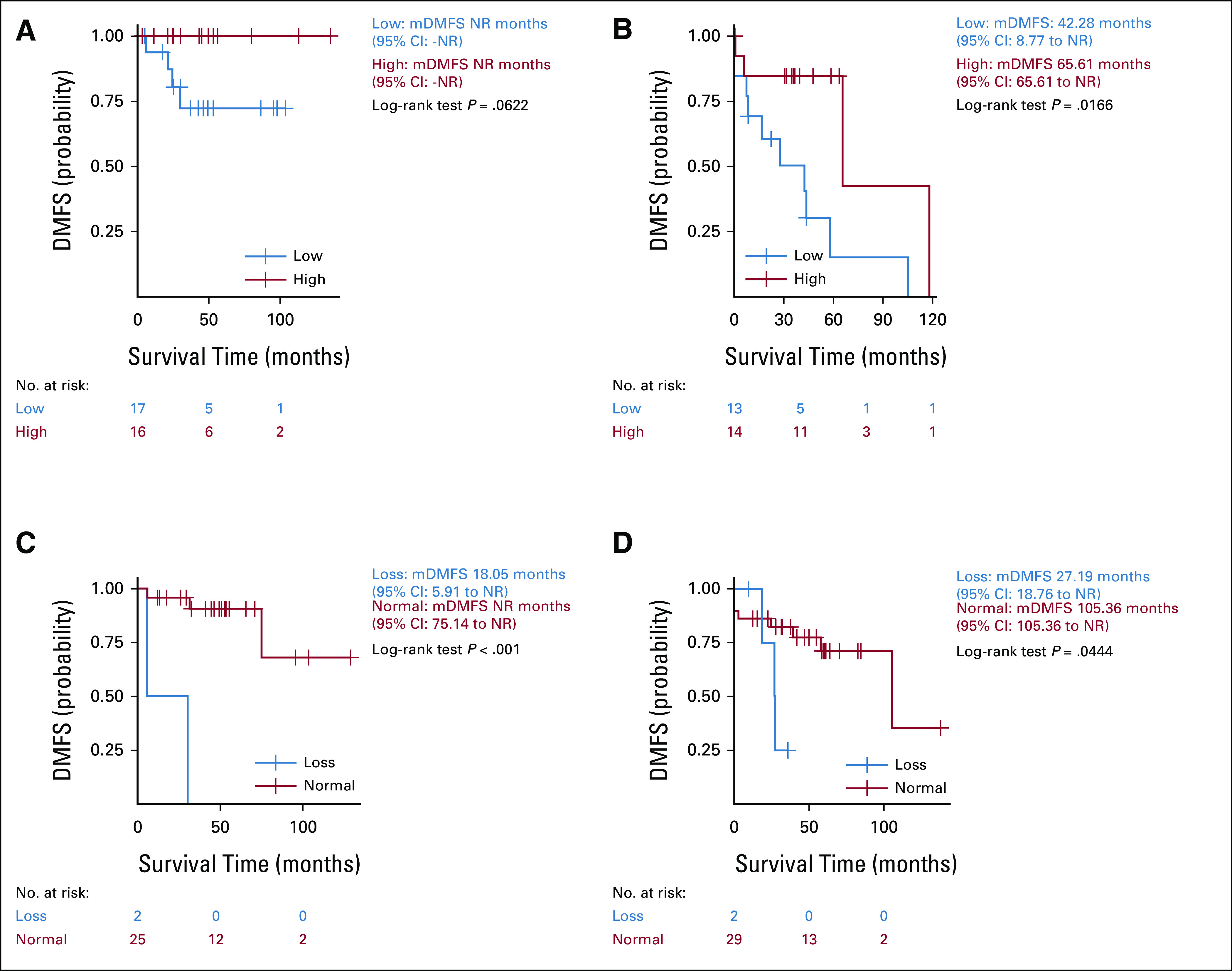
Low expression or heterozygous loss of PTEN independently predicts poor survival in clinically low/intermediate or high groups: (A) DMFS: clinically low/intermediate group in the GIST-60 cohort, (B) DMFS: clinically high group in the GIST-60 cohort, (C) DMFS: clinically low/intermediate group in the GIST-100 cohort, and (D) DMFS: clinically high group in the GIST-100 cohort. GIST, gastrointestinal stromal tumor; DMFS, distant metastatic-free survival; NR, not reached; CI, confidence interval.

In GIST-60, patients with PTEN low expression had reduced DFS and DMFS compared with patients with PTEN high expression in patients clinically considered at low/intermediate risk (on the basis of AFIP/prognostic contour map); however, the difference was not identified as statistically significant (mDMFS: NR; *P* = .062). Larger sample size and longer FU would be required to accurately report the number of events (Fig 3A). In patients clinically considered at high risk and PTEN low expression, DFS and DMFS (mDMFS, 42.28 months; 95% CI, 8.77 to NR) were significantly reduced compared with those with PTEN high expression (mDMFS, 65.61 months; 95% CI, 65.61 to NR; *P* = .0166; Fig 3B). Similarly, in GIST-100, patients clinically considered at low/intermediate risk with PTEN heterozygous loss had reduced DFS and DMFS (mDMFS, 18.05 months; 95% CI, 5.91 to NR) than those with PTEN normal status (mDMFS, NR; 95% CI, 75.14 to NR; *P* < .001; Fig 3C). In patients clinically considered at high risk, PTEN heterozygous loss had reduced DFS and DMFS (mDMFS, 27.19 months; 95% CI, 18.76 to NR) than patients with PTEN normal status (mDMFS, 105.36 months; 95% CI, 105.36 to NR; *P* = .044 (Fig 3D).

## DISCUSSION

Our study showed that PTEN low expression and PTEN heterozygous loss are independent prognostic factors associated with an increased risk of relapse in localized GIST, which may complement the classically used clinical prognostic factors.

To date, several prognostic biomarkers have been investigated in localized GIST besides RTKs (KIT, PDGFRA), such as cyclin-dependent kinase inhibitor 2A (CDKN2A/p16), aurora kinase A (AURKA), neurofibromatosis type 2 (NF2), histone modifier gene SET domain containing 2 (SETD2), Raf kinase inhibitor protein (RKIP), potassium channel tetramerization domain containing protein 10 (KCTD10), SLIT and NTRK-like family member 3 (SLITRK3), or orphan receptor 2 (ROR2).^31^ However, these prognostic biomarkers were mainly issued from studies with limited sample size. In addition, only few studies have investigated whether these biomarkers provide an added value to the current clinical prognostic tools. Therefore, although scientifically interesting, they have limited impact on routine clinical care in patients with GIST so far.

This study showed that PTEN low expression had a significant prognostic value in patients with localized GIST, which is consistent with previous results,^12,17-19^ but also identified PTEN loss as an independent prognostic factor that added value to the current standardized clinical prognostic tools. Our group previously reported that genomic index (GI) and complexity index for sarcoma (CINSARC) had significant prognostic value in localized GIST. These gene signatures, as a reflect of genomic instability, outperformed clinical prognostic tools such as AFIP.^28,29^ It would be interesting to further investigate the interaction and combined value of PTEN with these gene signatures and with some other prognostic biomarkers especially when targetable, such as PI3KCA and tuberous sclerosis 2 (TSC2) in PI3K/AKT/mTOR pathway, or alternatively pathways involved in cell cycle regulation (cyclin-dependent kinase 4 [CDK4], E2F1, and cyclin D2 [CCND2]) or in epigenetic regulation (eg, AURKA), which are known to be involved in tumor relapse and progression in GIST. Our initial analysis in GIST-60 cohort showed that these biomarkers have standalone prognostic value (data not shown), but much larger cohorts are required for validation of preliminary results and further investigation on the combined value of these biomarkers needs to be performed.

Overall, 40%-50% of the patients with localized GIST will develop metastases usually localized in the liver within the first 2-10 years after complete resection of primary tumor depending on the nature of disease as reflected by mitotic count and Ki-67.^2^ Adjuvant imatinib has consistently demonstrated significant efficacy in halving the risk of relapse in patients with localized GIST, especially in patients harboring drug-sensitive mutations; however, only patients clinically considered at high risk can be treated so far.^8^ Despite adjuvant treatment demonstrated efficacy, about 35% of the patients with localized GIST still recur.^8,32^ Our study showed that survival in clinically high-risk patients is worse than in low-/intermediate-risk patients, as expected (Fig 3). PTEN low expression and PTEN heterozygous loss are identified as independent factors associated with poor DMFS compared with patients with PTEN high expression and PTEN normal status (Figs 3C and 3D). Notwithstanding the limited sample size of the series, mDMFS for patients with PTEN heterozygous loss was only 27 months, ie, about four times shorter than mDMFS in patients with PTEN normal status (105 months), suggesting that specific adjuvant strategy such as combined therapies involving TKIs and drugs specifically targeting PI3K/AKT/mTOR pathways should be developed to improve their clinical outcome. On the basis of potential signal of efficacy of the treatment combining imatinib and the mTOR inhibitor everolimus in unselected advanced GIST patients,^25^ PTEN loss may be useful as selection stratification criteria to refine design of future adjuvant and/or advanced clinical trial. Other drug combined with TKIs could also be considered such as AKT inhibitors and/or RAF/MEK inhibitors because of negative feedback activation of MAPK pathway.^20,21^ In addition, among the patients clinically considered at low/intermediate risk, approximately 5%-20% of these patients will relapse with or without imatinib, considering that adjuvant imatinib is currently not used as standard treatment.^2-7,9,10^ Generally, only surveillance with regular image scans is required according to local jurisdictions. Our study demonstrated that patients with PTEN low expression or PTEN heterozygous loss had worse clinical outcome, although they are not identified as patients at high risk. For example, mDMFS was only 18 months in patients with PTEN heterozygous loss, which is unusual on the basis of clinical prognostic tools, versus NR in patients with PTEN normal status because of relatively short FU duration of just over 4 years (Figs 3A and 3B). These data indicate that patients with PTEN loss should undergo more stringent surveillance and close monitoring to allow early detection of potential recurrent/metastatic disease and be able to initiate aggressive systemic treatments combined with locoregional treatments (ie, metastectomy or other ablative therapies) and to improve overall clinical outcome.

Despite the sample size of our study (n = 160) was relatively larger compared with series on PTEN biomarker previously published in GIST (n = 20-104), to the best of our knowledge,^12,17-19^ our sample size was nevertheless limited and faced with reduced event rate, and wide CI is observed in most survival analysis. In addition, it may explain why PTEN lower expression numerically predicted poorer DFS and DMFS in low-/intermediate-risk group but did not reach statistical significance (data not shown; Fig 3A). Further validation of our results would be required using an independent large cohort, and complementary approaches such as immunohistochemistry to assess PTEN expression on tissue microarray may be warranted. Immunohistochemistry would also be more easily implemented into routine clinical practice. Another limitation of this study is that 38% of patients' clinical/tumor characteristics information is missing in GIST-100, which prevent multivariate analysis to be performed in GIST-100. Instead, Fisher's exact test was used and revealed that *PTEN* loss was significantly associated with local or metastatic recurrence (*P* = .0001; data not shown). Finally, the added prognostic value of PTEN on overall survival cannot be accurately estimated because of the limited FU duration and reduced event rate in both cohorts, and prevent to appropriately determine mDFS, mDMFS, and related confidence intervals.

In conclusion, to our knowledge, our study is one of the few studies investigating the prognostic role of PTEN biomarker, PTEN low expression, or PTEN loss in the context of standardized clinical prognostic tools. PTEN low expression and PTEN loss consistently and independently predicted poorer survival not only in clinically high-risk patients but also in patients at low/intermediate risk, prompting further development of therapeutic and surveillance strategies to improve their clinical outcome. Clinically low-/intermediate-risk GIST patients with PTEN low expression/PTEN loss may benefit from intensive surveillance, whereas clinically high-risk GIST patients with PTEN low expression/PTEN loss may benefit from further clinical trials to investigate the additional value of mTOR inhibitors to standard adjuvant TKIs. Our study strongly suggests that PTEN loss strengthens the prognostic value of standardized clinical prognostic tools and warrant to be considered for implementation besides standardized clinical prognostic tools to further guide clinical management of patients with localized GIST.

## Data Availability

http://atg-sarc.sarcomabcb.org/; on-demand access
